# Experience of a vascular ultrasound-guided program: from the ICU to the hospital

**DOI:** 10.1186/s13089-024-00393-2

**Published:** 2024-09-19

**Authors:** Miguel Angel Oviedo-Torres, Andrés Felipe Yepes-Velasco, Jeimy Lorena Moreno-Araque, David Rene Rodríguez-Lima, Andrés Felipe Mora-Salamanca

**Affiliations:** 1 Department of Critical Medicine and Intensive Care, Fundación Santa Fe de Bogotá, Carrera 7 No. 117 – 15, Bogotá, Colombia; 2 School of Medicine and Health Sciences, Universidad del Rosario, Bogotá, Colombia

**Keywords:** Peripherally inserted central catheter line insertion, Central venous catheters, Catheter-related infections, Venous thrombosis, Complications, Incidence, Odds ratio, Colombia

## Abstract

**Background:**

The use of peripherally inserted central venous catheters (PICCs) has increased worldwide in the last decade. However, PICCs are associated to catheter-related thrombosis (CRT) and central line-associated bloodstream infections (CLABSIs). We describe the characteristics of patients requiring a PICC, estimate the incidence rate, and identify potential risk factors of PICC-related complications.

**Methods:**

All adult patients requiring a PICC at our institution (Fundación Santa Fe de Bogotá, Bogota, Colombia) from September 2022 to May 2024 were included in the analysis. The database from active PICC monitoring collected demographic and PICC-related information. The incidence rate of CLABSI and CRT, and crude odds ratios (cORs) were estimated.

**Results:**

Overall, 1936 individuals were included in the study. The median age was 67 years (IQR: 50–78 years), and 51.5% were females. The median duration of PICC lines was 10 days (IQR: 4–17). Seventy-nine patients had catheter-related complications, mostly in the Intensive Care Unit (ICU). The CLABSI and CRT institutional incidence rates per 1000 catheter-days were 2.03 (2.96 in the ICU) and 0.58 (0.61 in the ICU), respectively. Prolonged catheter use (≥ 6 days), PICC insertion in the intensive care unit, and postoperative care after cardiac surgery were identified as potential risk factors for CLABSI, while a catheter insertion into the brachial vein was associated with CRT.

**Conclusion:**

Daily PICC assessment, particularly in patients with prolonged catheter use, PICC insertion into the brachial vein, or in postoperative care after cardiac surgery may significantly reduce CLABSI and CRT cases. Implementing Vascular Access Teams, venous catheter care bundles, and institutional insertion protocols optimize clinical outcomes.

**Supplementary Information:**

The online version contains supplementary material available at 10.1186/s13089-024-00393-2.

## Background

Vascular access is key in the clinical management of nearly all patients attending healthcare facilities, particularly in inpatient and Intensive Care Unit (ICU) settings. Among the diversity of catheterization options, peripherally inserted central catheters (PICCs), a subset of central venous catheters (CVCs), are an appealing option for oncological patients, those with limited peripheral access, and ICU patients. Unlike other CVCs, PICCs are easier to insert and have fewer morbidity complications [1, 2]. Similarly, compared to peripheral venous catheters, PICCs can be used to administer irritant, hyperosmolar, and extreme pH drugs or solutions, and medications for several weeks or months. Despite their advantages, PICCs are not exempt from complications, with catheter-related thrombosis (CRT) and central line-associated bloodstream infections (CLABSIs) being the most common [3]. In this study, we would like to share our experience inserting PICC lines in our institution by describing the characteristics of adult patients requiring a PICC, estimating the incidence rate, and identifying potential risk factors for PICC-related complications in our institution from September 2022 to May 2024.

## Methods

### Data

All adult patients (≥ 18 years) requiring a PICC at our institution (Fundación Santa Fe de Bogotá, Bogota, Colombia) were included in the analysis. Data were obtained from an institutional database that monitors all PICCs inserted in both the inpatient and outpatient settings. This database collects demographic and PICC-related information, including catheter and vein diameter, site of catheter insertion, number of venipuncture attempts, catheter days, PICC-related complications, and indications for PICC removal. CLABSI were identified according to the Centers for Disease Control and Prevention definition [4]. CRT included both superficial and deep vein venous thrombosis cases. The data are gathered by the Institutional Vascular Access Team—an ICU initiative formulated as a standard of care—which has been extended to the whole institution. The team includes physicians and nurses trained in PICC insertion using ultrasound guidance.

#### PICC insertion

The PICC insertion technique is based on the recommendations of the Safe Insertion of PICCs (SIP) protocol [5], which includes the following steps:

1. Patient Preparation: The procedure is explained to the patient and informed consent is obtained.
2. Vein Selection: A suitable vein is selected for catheter insertion, usually in the arm. The basilic vein is preferred, but the brachial vein may be used if the basilic vein’s diameter is less than 3 mm.
3. Ultrasound Scanning: Venous flow is confirmed through pulsed Doppler.
4. Catheter Size: A 1 Fr catheter corresponds to a diameter of approximately 0.33 mm. Therefore, a 3 Fr catheter has a diameter of about 1 mm, a 4 Fr catheter about 1.33 mm, and so on. Ideally, the catheter to vein diameter ratio should be 1:3 (33%).
5. Skin Disinfection: The skin at the insertion site is disinfected with a 2% chlorhexidine solution.
6. Catheter Insertion: Guided by ultrasound to minimize the risk of complications.
7. Measurement of Catheter Insertion Distance: From the puncture site to the mid-clavicular line, add 10 cm if on the right side, 13 cm if on the left.
8. Advancement of the Catheter: The catheter is advanced to a central vein such as the superior vena cava.
9. Confirmation of Catheter Position: The final catheter tip position is confirmed through two safety checks involving the subclavian and internal jugular veins.
10. Catheter Fixation: Using sutureless stabilization devices, the catheter is secured, and the exit site protected.
11. Follow-up: Monitored daily by the vascular access group, noting any early signs of local infection or potential complications.

**Fig. 1:**
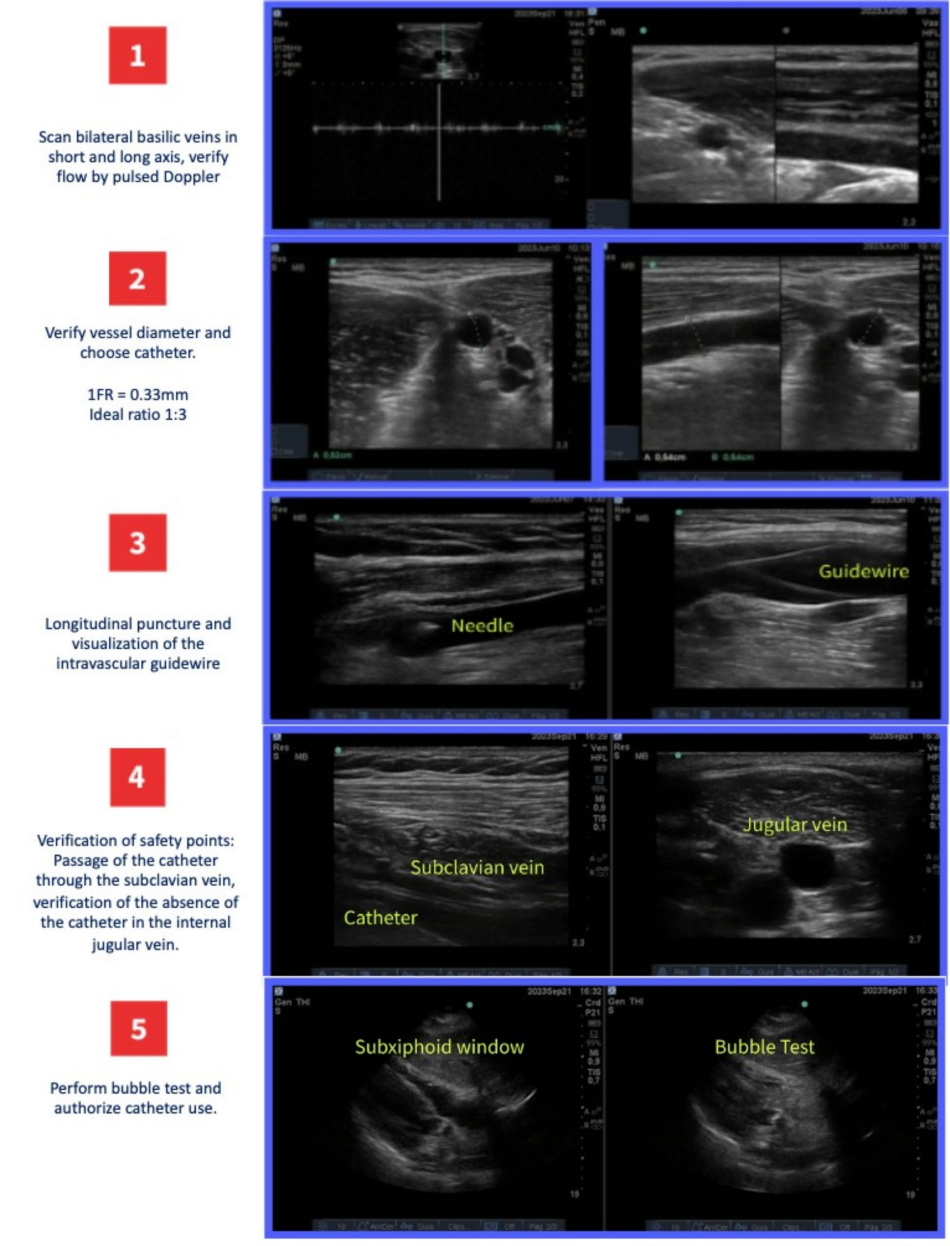
Steps for the insertion of a peripherally inserted central venous catheter using the ultrasonographic technique

These steps may vary slightly depending on specific hospital practices and individual patient needs (see Fig 1).

### Statistical analysis

Categorical variables were reported as frequencies and proportions. The normality of continuous variables was assessed using the Shapiro-Wilk test. Normally distributed variables are presented as means and standard deviations, and non-normally distributed variables as medians and interquartile ranges. The incidence rate of catheter-related complications was estimated for each complication (CLABSI, CRT, malposition/migration, malfunction, and occlusion) using the total number of patient days (catheter days) as the denominator. Crude odds ratios (cORs) and their 95% confidence intervals (CI) were estimated after performing a univariate analysis using the chi-square test. Statistical analyses were performed using STATA 17.0 (College Station, Texas, USA).

## Results

Between September 2022 and May 2024, 1936 PICCs were inserted in our institution. The patients’ median age was 67 years (IQR: 50–78 years), and the proportion of female patients (51.5%) was slightly higher than that of male patients. Almost 40% of patients were diagnosed with sepsis, followed by cerebrovascular disease (10.7%), and most PICC lines were inserted in the ICU (57.7%). The main indication for PICC insertion was the administration of vesicant/irritant drugs (66.7%). Seventy-nine patients had catheter-related complications, with CLABSI and CRT being the most common. Thirty-nine out of fifty-nine CLABSI cases and half of the CRT cases were reported in the ICU. The median duration of the PICC lines was 10 days (IQR: 4–17) (Table 1).

The CLABSI incidence rate per 1000 catheter-days was 2.03 at the institutional level and 2.96 in the ICU. The CRT incidence rates were lower compared to CLABSI, at 0.58 and 0.61 per 1000 catheter-days at the institutional and ICU levels, respectively.

Evidence from these data shows that patients with prolonged catheter use (≥ 19 days) are 34.2 times more likely (95% CI: 4.60-254.4; p-value = 0.001) to develop a CLABSI than patients with short-term catheter use (0–5 days). Likewise, patients with a catheter use between 6 and 10 days and those between 11 and 18 days are 12.4 (95% CI: 1.57–98.5; p-value = 0.02) and 26.7 (95% CI: 3.56–200.1; p-value = 0.001) more likely to develop a CLABSI than patients with short-term catheter use, respectively.

Additionally, patients in postoperative care after cardiac surgery are 6.61 times more likely (95% CI: 2.62–16.7; p-value = < 0.001) to develop a CLABSI than patients diagnosed with sepsis. PICC insertion in the ICU is more likely to develop a CLABSI compared to insertion in the general ward or the emergency room, while patients 80 years old and over are less likely to develop a CLABSI than patients between 18 and 39 years (Table 2).

On the contrary, no associations were identified between demographic, clinical, and PICC-related variables, and CRT. There is evidence (cOR: 3.42; 95% CI: 1.16–10.1; p-value = 0.03) suggesting that patients with PICCs inserted into the brachial vein are more likely to develop CRT than patients with PICCs inserted into the basilic vein (Table 1).

**Table 1:** Demographic and clinical characteristics of patients requiring a peripherally inserted central venous catheter

Variables	*N* (%)
Age (median, IQR, years)	67 (50–78)
Female	997 (51.5)
Main diagnosis	
Sepsis	766 (39.6)
Cerebrovascular disease	207 (10.7)
Burn	84 (4.3)
Heart failure	59 (3.1)
Cardiovascular surgery	25 (1.3)
Other	795 (41.1)
PICC insertion department	
Intensive Care Unit	1051 (54.3)
General ward	678 (35.0)
Emergency room	207 (10.7)
PICC indication	
Administration vesicant/irritating drugs	1292 (66.7)
Total parenteral nutrition	324 (16.7)
Limited peripheral access	238 (12.3)
Catheter change	64 (3.3)
HAHP	18 (0.9)
Catheter Size (Fr)*	
4	370 (19.1)
4.5	158 (8.2)
5	1139 (58.8)
5.5	268 (13.8)
Vein diameter (mm)	
Basilic vein	4 (3.8–4.3)
Brachial vein	4 (3.7–4.2)
Cephalic vein	3.55 (3.3–3.8)
CVR% (median, IQR, %)	17 (14.1–21)
Site of catheter	
Basilic vein	1677 (86.6)
Brachial vein	249 (12.9)
Cephalic vein	10 (0.5)
Side of catheter	
Right	1150 (59.4)
Left	786 (40.6)
Catheter complication (*n* = 79)	
CLABSI	52
CRT	15
Other	12
Number of venipuncture attempts	
1	1912 (98.8)
2	19 (1.0)
3	5 (0.3)
Catheter days (median, IQR, days)	10 (4–17)
Indications for PICC removal	
Hospital discharge	986 (51.2)
No longer required	324 (16.8)
Death	231 (12.0)
HAHP	115 (6.0)
Transference to another institution	91 (4.7)
Catheter complication	79 (4.1)
Catheter-related problem	70 (3.6)
Accidental self-removal	30 (1.6)

*One case required a 6 French catheter size

CLABSI: central line-associated bloodstream infections; CRT: catheter related thrombosis; CVR: catheter-to-vein ratio; IQR: Interquartile range; HAHP: hospital at home program; PICC: peripherally inserted central venous catheter

**Table 2:** Crude odds ratios for the association between catheter-related complications (CLABSI and CRT) and demographic, clinical and PICC-related characteristics

Variable	CLABSI			CRT		
Category	cOR	95% CI	p-value	cOR	95% CI	p-value
Age (years)						
10–39 40–59 60–79 ≥ 80	1 1.67 1.22 0.78	- 0.32–8.72 0.25–5.95 0.11–5.62	- 0.54 0.80 0.80	1 0.99 1.4 0.39	- 0.16–6.02 0.29–6.67 0.03–4.33	- 0.99 0.67 0.44
Sex						
Female	1	-	-	1	-	-
Male	1.86	0.67–5.16	0.23	0.83	0.28–2.40	0.73
Main diagnosis						
Sepsis Cerebrovascular disease Cardiovascular surgery Heart failure Burn Other	1 3.35 5.65 2.75 4.84 2.15	- 0.74–15.2 0.60–53.2 0.30–25.2 0.86–27.2 0.64–7.21	- 0.12 0.13 0.37 0.07 0.21	1 0.43 2.23 1.08 0.93 0.42	- 0.05–3.41 0.27–18.4 0.13–8.70 0.12–7.46 0.13–1.35	- 0.43 0.46 0.94 0.95 0.15
PICC insertion department						
Intensive Care Unit Emergency room General ward	1 0.49 0.26	- 0.06–3.83 0.06–1.18	- 0.50 0.08	1 2.84 1.00	- 0.72–11.2 0.29–3.43	- 0.14 0.99
PICC indication						
Limited venous access Catheter change Irritant drugs Parenteral nutrition HAHP	1 15.9 3.98 3.23 12.3	- 1.61–157 0.51-31.0 0.33–31.3 0.73–205	- 0.02 0.19 0.31 0.08	1 0.98 0.64 0.42 2.40	- 0.11–8.66 0.21–1.92 0.08–2.19 0.26–21.8	- 0.99 0.42 0.30 0.44
CVR (%)						
0.4–14.9 ≥ 15.0	1 0.98	- 0.87–1.10	- 0.75	1 1.11	- 0.99–1.26	- 0.08
Site of catheter						
Basilic vein Brachial vein	1 3.61	- 1.34–9.72	- 0.01	1 1.41	- 0.44–4.55	- 0.56
Side of catheter						
Right Left	1 1.06	- 0.39–2.87	- 0.91	1 0.75	- 0.25–2.27	- 0.62
Catheter days						
0–5 6–10 11–18 ≥ 19	1 1.18 7.62 9.72	- 0.07–18.9 0.91–63.8 1.21–78.3	- 0.91 0.06 0.03	1 1.17 1.67 1.58	- 0.24–5.88 0.37–7.52 0.35–7.14	- 0.84 0.51 0.55

CI: confidence interval; CLABSI: central line-associated bloodstream infections; cOR: crude odds ratio; CRT: catheter related thrombosis; CVR: catheter-to-vein ratio; HAHP: hospital at home program; PICC: peripherally inserted central venous catheter

## Discussion

Central venous access is crucial in various scenarios and for multiple purposes, such as hemodynamic monitoring, administering medications with acidic or alkaline solutions (e.g., antibiotics), providing vasopressor support, or using hyperosmolar solutions via parenteral routes that exceed 800 mOsm/L [6, 7]. , however, CVCs have been associated with potentially fatal complications such as pneumothorax, bloodstream infections, and thrombosis [8]. Over the last 10 years, the practice of inserting PICCs has evolved, some evidence is controversial regarding complications related to PICC catheters versus CVCs, even so, it has been demonstrated that following good practices for inserting peripherally inserted central venous catheters reduces complications related to thrombotic events or CLASBI compared to central venous catheters [9]. Reports indicate a reduction in complications associated with central catheter insertion after implementing vascular access teams [10, 11]. In a survey designed to assess perceptions and decision-making patterns regarding vascular access in ICUs of 13 centers, it was found that evidence-based practices are followed inconsistently and vary according to the device, training status, ICU situation, and hospital size. Additionally, 59% of the centers did not have written guidelines or protocols on the appropriate type of vascular access for ICU patients. Likewise, having local vascular access guidelines and protocols was associated with improved adherence to certain evidence-based practices [12]. It has also been suggested that the use of PICC over CVC reduces costs for specialized vascular access equipment [13], highlighting the importance of generating institutional groups specialized in the insertion, follow-up, and active monitoring of vascular access. This aligns with our findings, and to our knowledge, our cohort is the largest related to an ultrasound-guided vascular access team trained from the ICU to support the entire hospital.

Our study shows a low incidence rate of PICC-related complications (CLABSI and CRT) and the risk factors related to these complications. Considering CRT, a meta-analysis aimed at identifying the incidence of thromboembolic events associated with the use of PICC in hospitalized patients, including those in the ICU, found an incidence of 3.7% of symptomatic deep vein thrombosis, with a higher incidence in critically ill patients (10.6%) [14]. CRT is associated with the catheter diameter ratio, the size of the catheter, and the location of the central line tip. A lower catheter-to-vein ratio and a smaller catheter diameter result in reduced impact on vein flow, decreasing the subsequent risk of thrombosis. In our practice, a catheter-to-vein ratio < 33% is encouraged (262 patients in our study surpassed that limit). In addition, implementing general ward and ICU protocols (Additional file 2: Fig. S2, Additional file 3: Fig. S3) to choose venous access devices might explain our findings. However, it should be noted that in our group, we do not actively search for asymptomatic thrombosis, which has been shown to have higher incidences in patients with PICCs, especially in superficial veins, particularly in the first 2 weeks after device insertion. It is also identified as a risk factor for CRT the low venous flow rates, which, as mentioned previously, is closely related to the catheter-vein ratio, and in most of our patients had a ratio of less than 1:3. We found no differences between left vs. right basilic vein puncture as a risk factor, which has been previously described as a risk factor for asymptomatic CRT (meta-analysis of asymptomatic DVT) [3]. Nevertheless, adherence to pharmacological antithrombotic prophylaxis could modify these results, the data of which are beyond the scope of this study. The adoption of evidence-based interventions, such as ultrasound-guided vein puncture [15], micro-introducers, novel materials, and sutureless securement devices [16] has also been shown to reduce PICC-related complications.

Regarding CLABSI previous evidence, a meta-analysis reported that patients with a PICC line have a lower CLABSI incidence (2.12 per 1000 catheter days) than those with CVCs (4.09 per 1000 catheter days), indicating a 48% lower risk of CLABSI in patients with PICCs [9]. We report similar incidence of CLABSI cases, most occurring in the ICU. Our findings might be attributed to daily follow-up strategies, which actively monitored all vascular accesses to identify early signs of local infection and constantly reassessed the catheter need.

The median number of catheter days was 10, which aligns with the Michigan Appropriateness Guide for Intravenous Catheters [17]. This guide recommends that PICC catheters should not be used in patients with a predicted duration of use below 6 days unless there are no other suitable vascular access options. Among the 605 patients with a PICC duration of less than 6 days, 72.9% were patients who were discharged, transferred to another institution, or died. The remaining patients required the PICC due to vesicant/irritant medications or parenteral nutritional administration or had limited vascular access.

The generalizability of these results is subject to certain limitations. For instance, the number of complications was low, which made unfeasible to perform a logistic regression model. Therefore, it is unknown if the identified risk factors are indeed risk factors or confounders, mediators, or effect modifiers. A longer study period or a multicentric study including a larger number of patients is required to perform multivariate analyses.

This study contributes to the safety profile evidence regarding the insertion and maintenance of PICCs. Following standard insertion guidelines, daily catheter routine care, and timely PICC removal should be implemented in institutions—particularly in ICUs—that regularly use PICCs to reduce the frequency of catheter changes and, most importantly, the number of catheter-related complications.

## Electronic supplementary material

Below is the link to the electronic supplementary material.

Supplementary Material 1

Supplementary Material 2

## Data Availability

The datasets used and analyzed during the current study are available from the corresponding author on reasonable request.

## Abbreviations

CI: Confidence interval
CLABSI: Central line-associated bloodstream infections
cOR: Crude odds ratio
CRT: Catheter related thrombosis
CVC: Central venous catheter
ICU: Intensive care unit
IQR: Interquartile range
PICC: Peripherally inserted central catheter
SIP: Safe Insertion of PICCs
