# Supplementary material for: Experience of a vascular ultrasound-guided program: from the ICU to the hospital
Source: Ultrasound J. 2024 Sep 19;16:43. doi: 10.1186/s13089-024-00393-2 (PMC11413268; doi:10.1186/s13089-024-00393-2)
Supplement: Supplementary file 1 — Supplementary Material 1 [file 13089_2024_393_MOESM1_ESM.docx]

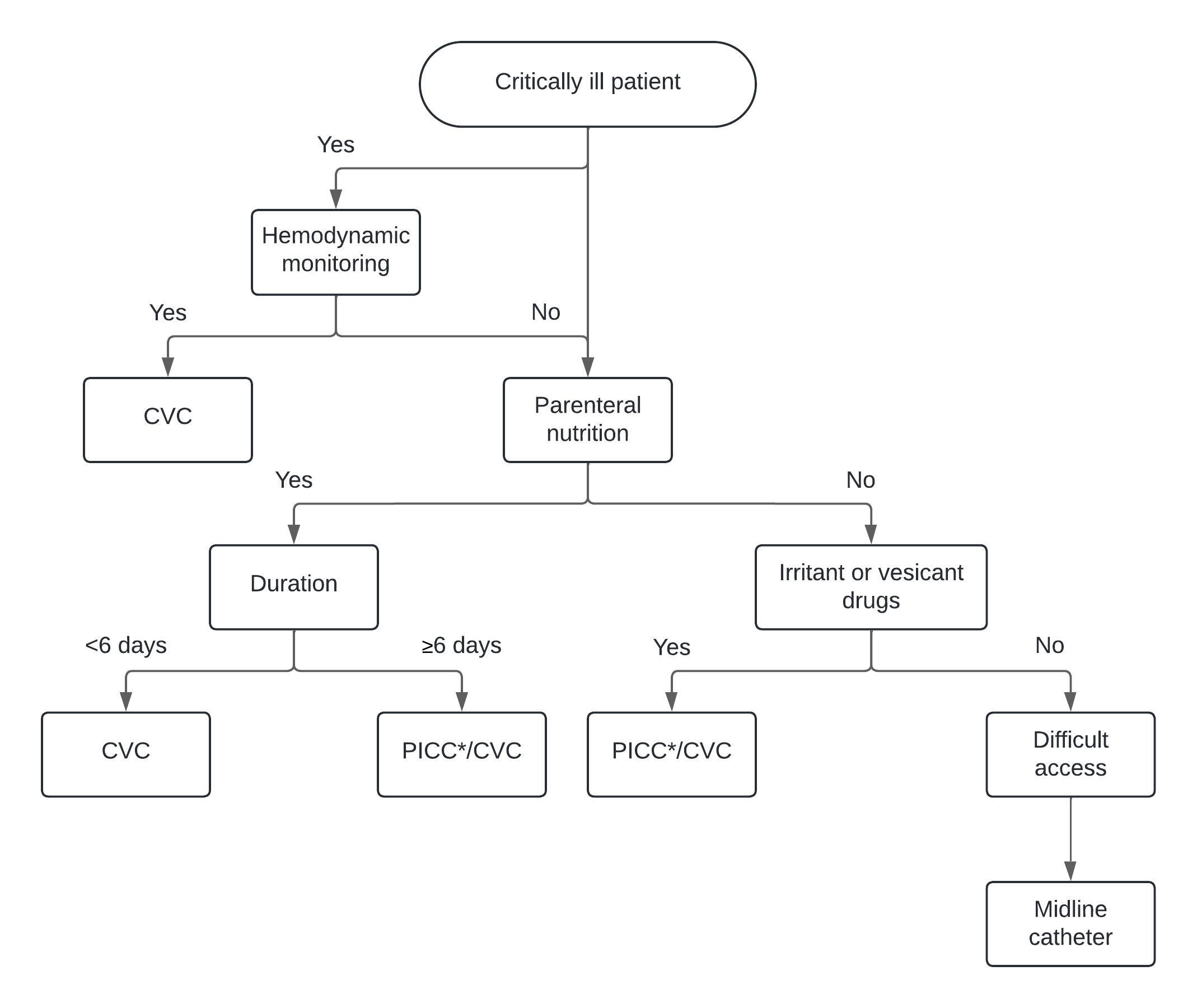


**Fig. S2.** Pathway for central vascular access in the intensive care unit. *Consider use of PICC according to the operator’s experience. CVC: Central venous catheter; PICC: peripherally inserted central venous catheter.
